# Adverse fetomaternal outcome among pregnant overweight women

**DOI:** 10.12669/pjms.312.6530

**Published:** 2015

**Authors:** Shazia Awan, Seema Bibi, Asadullah Makhdoom, Sumaiya Farooq, Tahir SM, Roshan Ara Qazi

**Affiliations:** 1 Shazia Awan, Research Associate, Dept. of Gynae & Obstretics, Liaquat University of Medical & Health Sciences, Jamshoro, Sindh, Pakistan; 2 Dr. Seema Bibi, Associate Professor, Dept. of Gynae & Obstretics, Liaquat University of Medical & Health Sciences, Jamshoro, Sindh, Pakistan; 3 Asadullah Makhdoom, Assistant Professor, Department of Orthopaedic Surgery & Traumatology, Liaquat University of Medical & Health Sciences, Jamshoro, Sindh, Pakistan; 4 Dr. Sumaiya Farooq, Senior Registrar, Department of Gynae & Obstretics, Liaquat University of Medical & Health Sciences, Jamshoro, Sindh, Pakistan; 5 Syed Muhammad Tahir, Assistant Professor, Department of Burn Surgery, Liaquat University of Medical & Health Sciences, Jamshoro, Sindh, Pakistan; 6 Dr. Roshan Ara Qazi, Professor, Dept. of Gynae & Obstretics, Liaquat University of Medical & Health Sciences, Jamshoro, Sindh, Pakistan

**Keywords:** Pregnant Overweight Women, Outcome, Adverse Fetomaternal

## Abstract

**Objective:**

To compare the adverse fetometernal out come in overweight and normal weight pregnant women.

**Methods:**

This comparative cohort study was conducted from 1^st^ October 2010 to 30 September 2012. Total 200 gravid women 100 were overweight and 100 normal weight pregnant women with gestational age for 08-40 weeks were included. Women having BMI (25 – 29.9 Kg/m^2^) were measured overweight and included in group A and 100 women having normal BMI of 18.5 to 24.9 as controls were in-group B. Chi-square test was applied to compare the proportion of maternal and fetal outcomes. Significant P – value of < 0.05 was considered.

**Results:**

The age range was between 30 to 45 years with mean age of 30±4.1 years in both groups.

Overweight pregnant women had significantly high frequency of pre-eclampsia (27% versus 9% in controls), PIH (24% versus 8% in controls), gestational diabetes mellitus (22% versus 5% in controls), prolonged labour (4% versus 6% in controls), Caesarean section (44% versus 16% in controls), Wound infection (3% versus 2% in controls) and Postpartum Hemorrhage (5% versus 2% in controls). P-value < 0.001 was considered significance. Fetal complications in overweight pregnant women compared to controls i.e. Still birth (13% versus 2%), Early neonatal death (11% versus 1%), shoulder dystocia (5% versus 1%) and NICU admission (47% versus 10%). Results were statistically significant except shoulder dystocia.

**Conclusion:**

We conclude that the result of present study indicates obesity exerts deleterious effect, both on fetal and maternal outcome.

## INTRODUCTION

The obesity, among chronic medical disorder is the most prevalent condition throughout world.^1^ Prevalence of the obesity is highly variable among different population and depends on the age and sex.^2^ In pregnant women the rate of obesity is rising and also aggregating the level of its significance and its impact on obesity-related pregnancy problems.^3^ The pregnancy problems those associated with maternal obesity mostly affect the mother but also fetus, neonate and older child.^4^ Obesity adversely effects not only the chance of conception but also increases the risk of miscarriage and congenital anomalies and decreases the response of treatment to fertility^5^ in addition to possible adversarial effects on the health of mother and their infant and other complications like gestational diabetes, pregnancy induced hypertension, cesarean delivery, macrosomia & infections.^6^

The World Health Organization^7^ and the National Institutes of Health^8^ define the body mass index (BMI) less than 18.5 as underweight, 18.5–24.9 as normal weight, 25–29.9 overweight, and more then 30 BMI labeled as obesity. Obesity is more categorized by BMI in Class I (30 –34.9), Class II (35–39.9), and Class III (greater than 40).

The most common international method to measure the obesity is the body mass *index* (BMI), or *Quetelet index*.^9^,^10^ Maternal obesity is the obstetric problem that is related to maternal pre-gravid obesity^11^ instead of excessive weight gain during pregnancy. The difference between pre pregnancy weight and weight of woman at the time of labor can be considered weight gain in pregnancy.

Obesity is an increasing problem in Pakistan and especially in females due to their inactive lifestyle, irregular eating patterns and fat rich diet. Objective of this study was to find out the frequency of overweight pregnant women, and compare with the adverse fetomaternal out come in overweight and normal weight pregnant women.

## METHODS

This comparative cohort study was conducted from 1^st^ October 2010 to 30 September 2011. A total of 200 gravid women in first trimester were selected for the study. The sample size was calculated through the Raosoft software by using the proportion of 25% of all obstetrical admissions with 90% confidential level and 10% of margin of error. Out of 3090 obstetrical admissions 266 pregnant women were found to overweight making the frequency of 8.6%, the sample size stands to be n = 266. Sample technique was used by non-probability purposive consecutive after taking informed consent. Inclusion criteria were pregnant women with singleton pregnancies with gestational age for 08-40 weeks. The exclusion criteria, includes pregnant women with history of medical disorders like thyroid, renal, diabetes mellitus type II and adrenal disorders.

Demographic variables recorded included age and body weight measured in kg, height in cm and body mass index (BMI) was calculated by dividing weight (kg) from height in meter squared (m^2^).

BMI = weight in kilograms

(kg/m²) height in meters²

To categorize patients as normal, overweight and obese, cut off points suggested by the World Health Organization (WHO) were used as: Organization (WHO) were used as: Normal weight: BMI <24.9kg/m^2^; Overweight: BMI >25 to <29.9kg/m^2^; and Obesity: BMI >30kg/m^2^.

Women having BMI (25 – 29.9 Kg/m^2^) were considered overweight and recruited to group A. The 100 women having normal BMI between 18.5- 24.9 were included as controls (group B). All overweight patients during the study period could not be included due to drop out secondary to exclusion criteria, non-consent of the patient and loss of follow up.

All pregnant women were evaluated by history, clinical examination, and routine investigations and managed according to the protocol of ward. Outcome variables were maternal like pre-eclampsia, pregnancy induced hypertension, gestational diabetes mellitus, caesarean section, wound infection, labour complications like prolong labour & PPH and fetal were still birth, early neonatal death, fetal trauma, NICU admission and shoulder Dystocia.

All required information including age, parity, gestational age, BMI and feto-maternal complication was recorded on predesigned proforma by principal investigator. Results were prepared with help of tables and graphs.

The study was performed after the permission of ethical committee of hospital, and written informed consent for the study. Patients fulfilling the inclusion criteria enrolled for study.

Data was entered and analyzed by statistical software SPSS version 16.0. Frequency and percentage were computed for variables like maternal age, parity and gestational age. Chi-square test was applied to compare the proportion of maternal and fetal outcomes in both the groups. P–value of < 0.05 was considered significant.

## RESULTS

During the period of study, 3090 pregnant women were admitted in the Department for various Obstetrical reasons including labor. Out of which 266 women were found overweight making the frequency of overweight pregnant population as (8.6%). Out of 266 overweight, a cohort of 100 was recruited for further comparison with control group of 100 normal weight women. The age range was between 30 to 45 years with mean age of 30 ±4.1 years in both groups. Table-I shows percentage, distribution of parity in both groups. Frequency of primigravida and multigravida is approximately same in both the groups.

**Table-I:** Percentage distribution of parity in both groups.

Parity	Overweight pregnant women Group		Normal weight pregnant women Group	
	No. of Patients	%	No. of Patients	%
Primigravida	17	17%	21	21%
Multigravida	83	83%	79	79%
Total	100	100%	100	100%

Maternal outcome in overweight and normal weight pregnant women was shown in Table-II. The pre eclampsia was the commonest adverse maternal outcome and most of these underwent caesarean section making maternal outcome gloomier. Similarly pregnancy induced hypertension; prolonged labour and Caesarean section found more common in overweight pregnant women as compared to control. However the frequency of wound infection and postpartum haemorrhage was approximately same in both groups.

**Table-II:** Percentage distribution of adverse maternal outcome in both groups.

Maternal outcome	Over weight Pregnant women (n=100)	Normal Pregnant women (n=100)	P Value†
Pre-eclampsia	27	9	0.0096*
Pregnancy induced Hypertension	24	8	0.0146*
Gestational diabetes mellitus	22	5	0.0041*
Prolong labour	4	6	0.7694
Caesarean section	44	16	0.0024*
Wound infection	3	2	0.9912
Post partum Hemorrhage	5	2	0.4653

† = Chi Square Test,

* = significant p-value

When considering fetal outcome, the stillbirth, early neonatal death and NICU admission were more frequent in overweight pregnant women as compared to controls. The commonest fetal morbidity seen in obese women was admission to neonatal intensive care unit, followed by still birth. The comparison of fetomaternal outcome in both is shown in Table-III.

**Table-III:** Percentage distribution of adverse Fetal outcome in both groups.

Fetal outcome	Over weight Pregnant women (n=100)	Normal weight Pregnant women (n=100)	P value†
Pre-eclampsia	27	9	0.0096*
Still birth	13	2	0.0133*
Early neonatal death	11	1	0.0121*
Shoulder dystocia	5	1	0.2321
NICU admission	47	10	<0.0001*

† = Chi Square Test,

* = significant p-value

## DISCUSSION

Increasing overweight influence the health of pregnant women, and also posses critical risk complications to maternal and Feto-Neonatal healthcare.^12^ The recent study of obesity carried out in United States of America, identifies that 28% of women under the age differences of 25 years or older are overweight, and, 27% are considered critically obese.^13^ The differences of frequency of overweight lies at 33%, Obese at 30%, lastly morbidly Obese, 4.5%.^14^

The present studies comprised of 17% Primigravida, 83% Multigravida, accordingly. The present study is comparable to Chaudhry H et al.^15^ According to the study conducted by Humaira, as stated above. It showed that, 30% patients were Primigravida and 70% were Multigravidas.^15^ The compatible mean age difference of the patients identified in the series was higher (30± SD4.1 Versus 26.4 Years) as compared to the study of Fatima S et al.^16^

Increased maternal BMI is one of the critical causes of risk factors of Preeclampsia. The comparative risk of pregnancy due to preeclampsia, is 35% higher with a mean comparative of BMI from 15 to 21. In addition to this, the rate is doubled and even tripled at changing BMI from 26 and 30.^17^ The hypothesis of inflammation and Hyperlipidemia, are also considered for the relative BMI preeclampsia.^18^ Increased BMI and Hyperlipidemia are some of the serious risk factors of preeclampsia, at this point, Hyperlipidemia may be a serious concern through which obesity causes Preeclampsia.

The present study identifies that 24% of cases were related to Pregnancy Induced Hypertension due to overweight; and relatively 8% cases of normal weight (P Value=0.0146). The findings are consistent and relevant to the other studies conducted at various occasions. It comparatively shows that Pregnancy Induced hypertension in obese women are 2.2 times higher as compared to control subjects.^19^

The risk of prolonged labor due to maternal obesity and extreme overweight is one of the common risk factor that usually comes under consideration in obstetric practice. The present study identifies Labor Complications, like prolong labor in 4% cases of overweight women and 6% cases of normal weight (P Value=0.7694). Contrasted to this, Arrowsmith S et al,^20^ reported that complications may increase to 30% of obese women. When a comparison is made between Normal and Morbidly obese women, the latter has significant higher rates of caesarian section.^21^

Hibbard et al.^22^ the present cases have encounter 44% of caesarian section in overweight women and 16% in normal weight. In another comparative study, it has been reported that 35% percent caesarian section occurred in Ngoga E et al^23^ Various studies have reported that increased risk of Post-Partum Haemorrhage occurrence in women that are obese or considerably overweight,^24^ 5% cases due to Post-Partum haemorrhage occurred in overweight women and 2% in normal weight, as it was identified in our study.

The present study has identified that in 100 cases of pregnant women that birth rate still counts 13% in overweight section and 2% in normal weight, where as (P Value =0.0133) in comparison to the other studies, 8% was in Fatima et al.^16^ Early neonatal death occurs in 11% of overweight and 1% of normal weight pregnant women (P Value=0.0121). Fetal Trauma was indentified in 5% cases in overweight women and 1% case occurrence in normal weight (P Value=0.2321). 47% cases of overweight and 10% cases of normal weight caused NICU admission (P Value=<0.0001).

## CONCLUSION

In conclusion, even moderate overweight has a significant deleterious effect on the outcome of pregnancy, and obesity leads to major maternal and fetal complications. In this study morbidly obese women were carefully ‘flagged’ as high-risk patients throughout their pregnancies. All attempts should be made to prevent obesity in women of childbearing age and to encourage weight loss before pregnancy. The consequences of obesity on maternal and fetal morbidity and mortality might be minimized through appropriate multidisciplinary management. We conclude that the results of the present study indicate obesity is associated with deleterious effect on fetomaternal outcome.
